# Supplementary figures and images for: Cross-site comparison of ribosomal depletion kits for Illumina RNAseq library construction
Source: BMC Genomics. 2018 Mar 15;19:199. doi: 10.1186/s12864-018-4585-1 (PMC6389247; doi:10.1186/s12864-018-4585-1)

## Slide 1
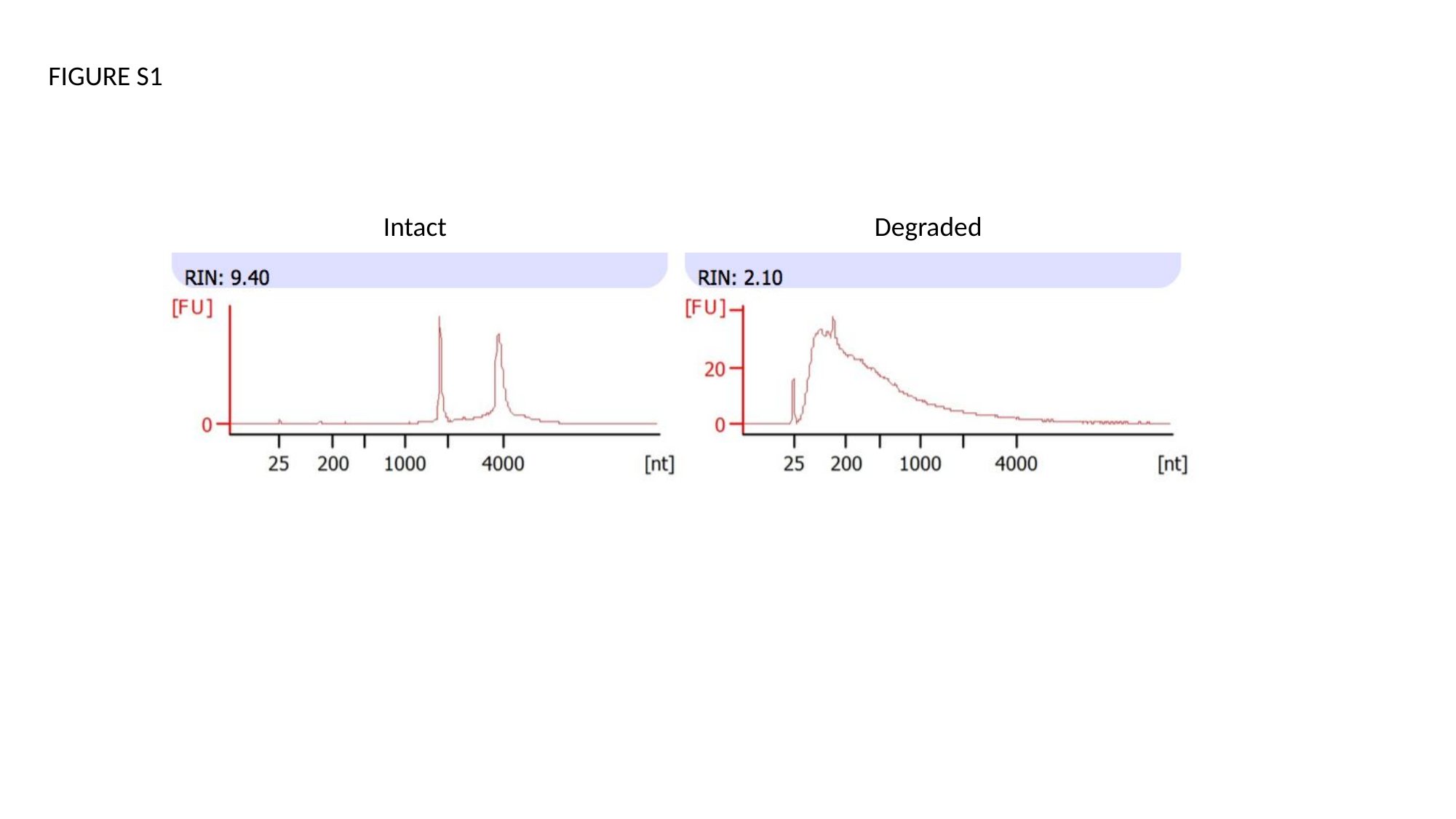

FIGURE S1
Intact
Degraded

Supplement: Supplementary file 1 — Figure S1. BioAnalyzer traces for samples used in the study. Left: Intact UHR, Right: Heat degraded UHR RNA. (PPTX 127 kb) [file 12864_2018_4585_MOESM1_ESM.pptx]

## Slide 1
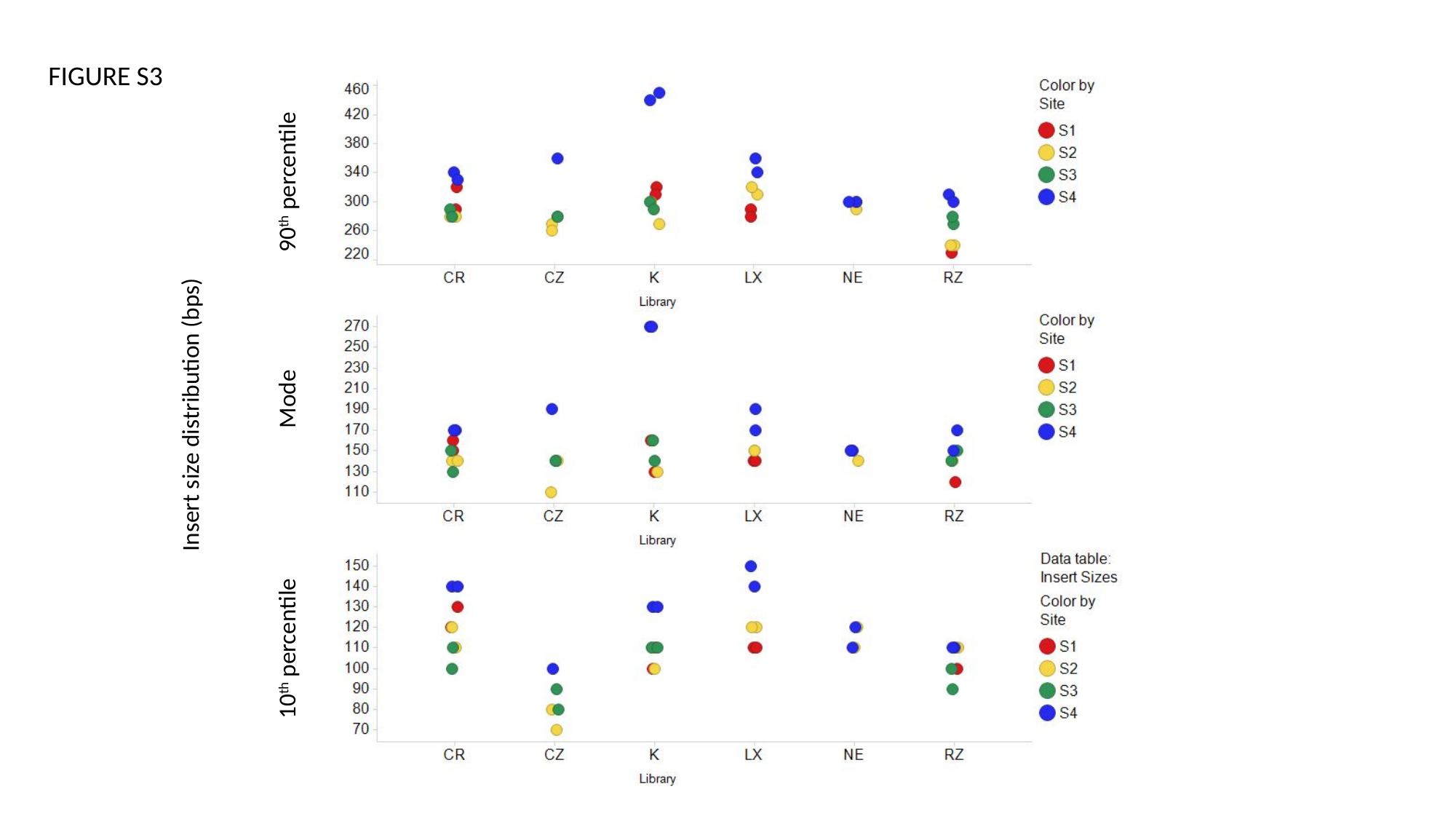

FIGURE S3
90th percentile
Mode
Insert size distribution (bps)
10th percentile

Supplement: Supplementary file 4 — Figure S3. Insert size distribution for RNAseq libraries from intact RNA. The insert size for each library passing the 50% rRNA filter was calculated for reads with convergent reads that were separated by < 1000 bp. Kit abbreviations: RZ = RiboZero Gold, LX = Lexogen RiboCop, NE = NEBNext rRNA Depletion, K=Kapa RiboErase, CR = Clontech Ribogone, CZ = SMARTer Pico total RNA. Top: length of the 90th percentile of inserts reads. Middle: length of the median insert read. Bottom: length of the 10th percentile of inserts read. (PPTX 82 kb) [file 12864_2018_4585_MOESM4_ESM.pptx]

## Slide 1
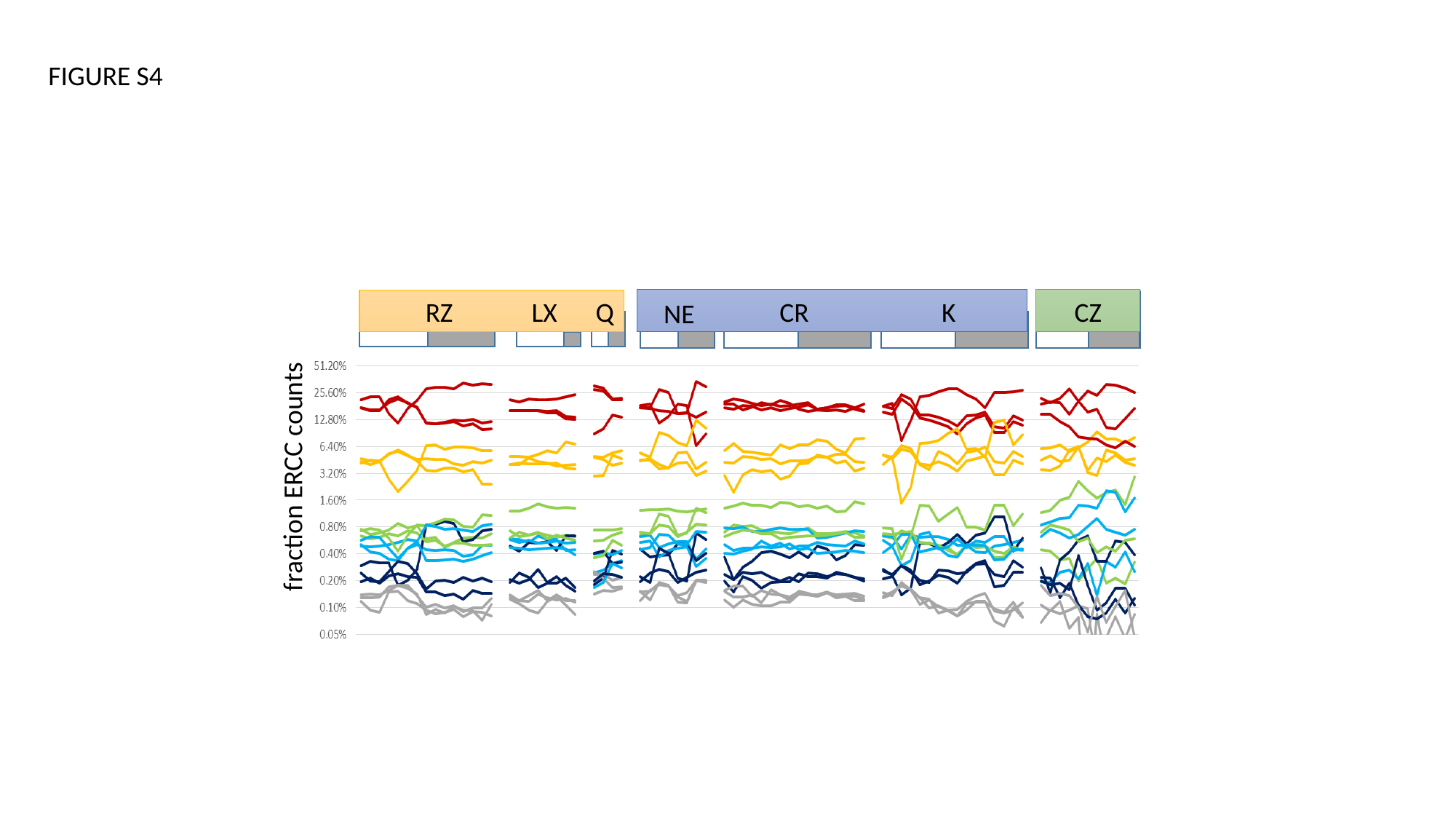

FIGURE S4
RZ
LX
Q
CR
K
CZ
NE
fraction ERCC counts

Supplement: Supplementary file 5 — Figure S4. Fraction of reads mapping to each ERCC mRNA is shown for each replicate. Light horizontal lines show 2-fold changes in fraction observed (log scale). Each expected concentration is shown in a different color. Data sets ordered by intact/degraded status followed by site within each kit left to right. (PPTX 165 kb) [file 12864_2018_4585_MOESM5_ESM.pptx]
