# Supplementary material for: Cross-site comparison of ribosomal depletion kits for Illumina RNAseq library construction
Source: BMC Genomics. 2018 Mar 15;19:199. doi: 10.1186/s12864-018-4585-1 (PMC6389247; doi:10.1186/s12864-018-4585-1)
Supplement: Supplementary file 2 — Contains the following: Describes normalization of kit protocols and any individual site deviations from this normalization. Figure S1. – BioAnalyzer traces for samples used in the study. Left: Intact UHR, Right: Heat degraded UHR RNA. Figure S2. – Clustering of differentially detected genes. Top 50 most differentially detected genes, as measured by variance of log2RPKM across all samples, were clustered based on their differential expression. TOP: Hierarchical tree of clustering based on a complete linkage function using Euclidean distance. 2ND LINE: Intact/Degraded status is shown. Intact samples are indicated in white while degraded samples are indicated in grey. 3RD LINE: Kit. Dark Blue = RZ|RiboZero Gold, Yellow = LX|Lexogen RiboCop, Aqua = NE|NEBNext rRNA Depletion, Green = Q|Qiagen, Grey = K|Kapa RiboErase, Blue = CR|Clontech Ribogone, Orange = CZ|SMARTer Pico total RNA. HEAT MAP: Red indicate higher level of absolute expression. Scale shown to right. White lines indicate the highest branches within the hierarchical tree. Figure S3. – Insert size distribution for RNAseq libraries from intact RNA. The insert size for each library passing the 50% rRNA filter was calculated for reads with convergent reads that were separated by < 1000 bp. Kit abbreviations: RZ = RiboZero Gold, LX = Lexogen RiboCop, NE = NEBNext rRNA Depletion, K=Kapa RiboErase, CR = Clontech Ribogone, CZ = SMARTer Pico total RNA. Top: length of the 90th percentile of inserts reads. Middle: length of the median insert read. Bottom: length of the 10th percentile of inserts read. Figure S4. - Relative ratio of reads mapping to ERCCs. Fraction of reads mapping to each ERCC mRNA is shown for each replicate. Light horizontal lines show 2-fold changes in fraction observed (log scale). Each expected concentration is shown in a different color. Data sets ordered by intact/degraded status followed by site within each kit left to right. Table S1. Catalog numbers and manual versions for protocols used. Tabl [file 12864_2018_4585_MOESM2_ESM.docx]

**Supplementary Methods**

Conference calls were conducted with each vendor and the sites using their respective kits. Fragmentation conditions for both the intact and degraded RNA samples were determined as well as the number of PCR cycles for each library kit used during the conference calls (Table S2). Additionally several vendors had recommendations due to differences in instrumentation between the sites.

Lexogen RiboCop Kit: Sites 2, 3, and 4 did not have access to a thermomixer. The vendor recommended using either a thermomixer without shaking for the incubation steps, or a thermalcycler without a heated lid and no mixing in all steps requiring a thermomixer. Kit performance did not appear to be affected by this modification as the lowest %rRNA was found for Site 4 using the thermalcycler. For all sites the ribo-depleted RNA was eluted with 8uL of nuclease-free water, 5uL of which was used for subsequent library prep with the NEBNext Ultra II Directional RNA Library Kit.

Qiagen GeneRead rRNA Depletion Nano kit: Site 3 did not have access to a thermomixer. The vendor recommended using a thermalcycler and vortexing every 5min during incubation of the depletion mix with the beads in place of the steps requiring a thermomixer. Kit performance did not appear to be adversely affected by this modification, the lowest %rRNA was found for site 3 using the thermalcycler and vortexing. For all sites the ribo-depleted RNA was eluted from an RNEasy MinElute Column with 10uL nuclease-free water, 5uL was used for subsequent library prep with the NEBNext Ultra II Directional RNA Library Kit.

Takara/Clontech RiboGone Kit: The ribo-depleted RNA was eluted with 10uL of nuclease-free water instead of RiboGone Purification buffer; 5uL was used for subsequent library prep with the NEBNext Ultra II Directional RNA Library Kit.

NEBNext Ultra II Directional RNA Library Kit: Regardless of rRNA depletion kit used (NEB, Lexogen, Qiagen or Takara/Clonetech RiboGone), 5uL of rRNA depleted RNA was used as input for library prep. The vendor recommended using the longer incubation time of 15 minutes in step 2.10.2 during Adapter Ligation Cleanup.

The remaining vendors specified RNA fragmentation conditions as well as PCR cycles, no other modifications from the provided protocols were noted.

**SUPPLEMENTARY TABLES**

**Table S1** – Catalog numbers and manual versions for protocols used.

| **Kit Name** | **Cat No** | **Manual/Protocol version** |
| --- | --- | --- |
| NEBNext rRNA Depletion Kit** | E7760 | 9/29/16 |
| Lexogen RiboCop** | 037.24 | 037UG073V0200 (V1.2) |
| Qiagen GeneRead rRNA Depletion Nano Kit** | 180224 | 09/2014 |
| Takara Clontech RiboGone Kit** | 634846 | 032814 |
| NEBNext Ultra II Directional RNA Library Prep | Early release | Early Access Protocol |
| KAPA RNA HyperPrep Kits with RiboErase (HMR) | KK8560 | KR1351 – v1.16 |
| Illumina TruSeq Stranded Total RNA kit with RiboZero Gold | RS-122-2303 | 15031048 Rev. E (LS protocol) |
| Takara Clontech SMARTer Pico kit | 635005 | 101215 |

**Table S2** – Comparison of RNA library preparation chemistry variables.

| Library kit | Fragmentation conditions  Intact RNA | Fragmentation conditions Degraded RNA | PCR cycles |
| --- | --- | --- | --- |
| NEBNext UltraII Directional RNA* | 15min, 94C | 5min, 94C | 12 |
| KAPA RNA HyperPrep kit | 6min, 94C | 1min, 65C | 16 |
| Illumina TruSeq Stranded total RNA | 8min | 1min | 15 |
| Takara Clontech SMARTer Pico kit | 4min, 94C |  | 15** |

* This library kit was used for 4 different rRNA depletion kits: NEBNext rRNA depletion, Lexogen RiboCop, Qiagen GeneRead rRNA Depletion kit, Takara Clontech RiboGone

* *For PCR2 step in manual
