# Supplementary material for: Cross-site comparison of ribosomal depletion kits for Illumina RNAseq library construction
Source: BMC Genomics. 2018 Mar 15;19:199. doi: 10.1186/s12864-018-4585-1 (PMC6389247; doi:10.1186/s12864-018-4585-1)
Supplement: Supplementary file 3 — Figure S2. Clustering of differentially detected genes. Top 50 most differentially detected genes, as measured by variance of log2RPKM across all samples, were clustered based on their differential expression. TOP: Hierarchical tree of clustering based on a complete linkage function using Euclidean distance. 2ND LINE: Intact/Degraded status is shown. Intact samples are indicated in white while degraded samples are indicated in grey. 3RD LINE: Kit. Dark Blue = RZ|RiboZero Gold, Yellow = LX|Lexogen RiboCop, Aqua = NE|NEBNext rRNA Depletion, Green = Q|Qiagen, Grey = K|Kapa RiboErase, Blue = CR|Clontech Ribogone, Orange = CZ|SMARTer Pico total RNA. HEAT MAP: Red indicate higher level of absolute expression. Scale shown to right. White lines indicate the highest branches within the hierarchical tree. (PPTX 179 kb) [file 12864_2018_4585_MOESM3_ESM.pptx]

## Slide 1
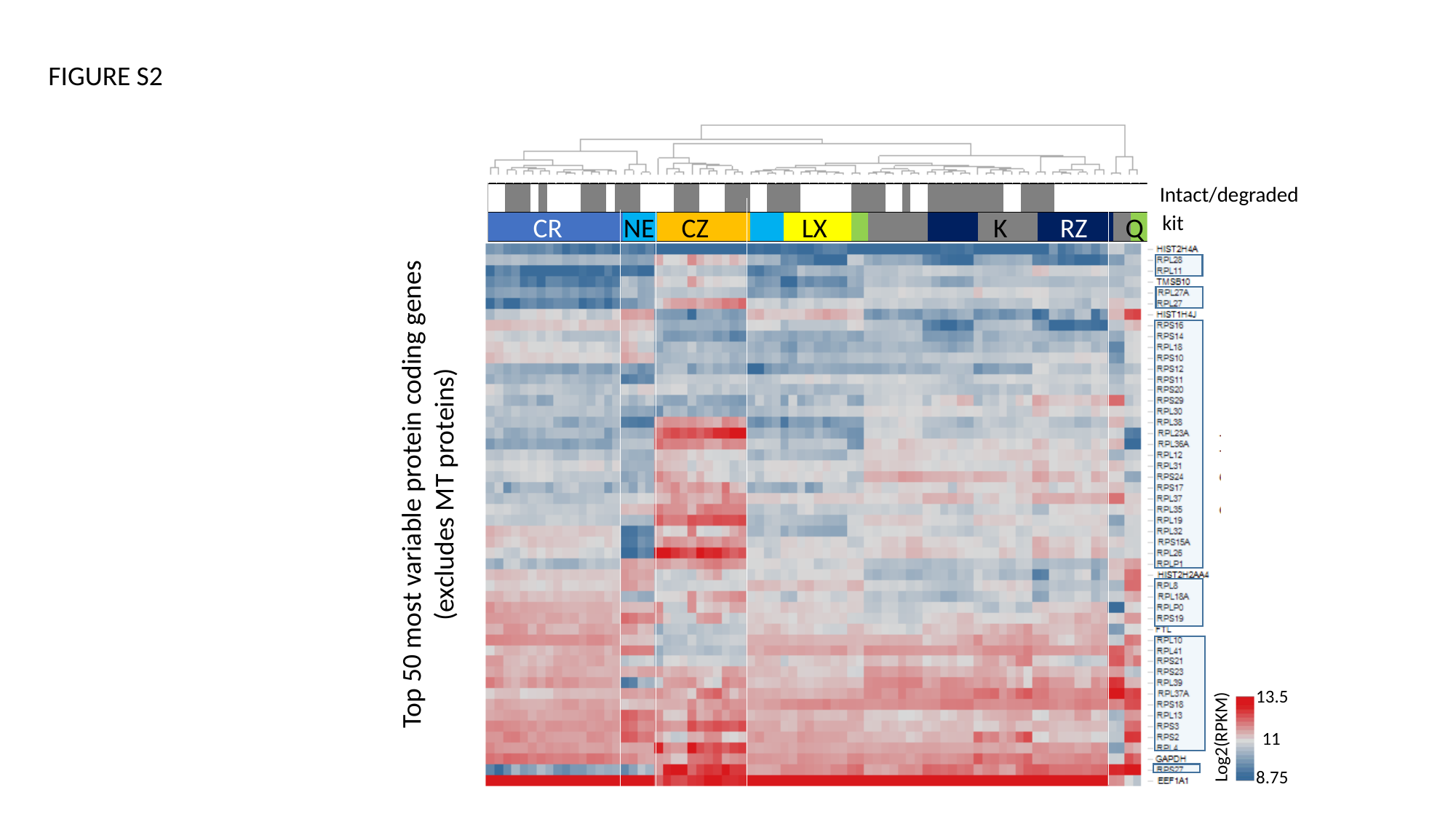

FIGURE S2
Intact/degraded
kit
CR
NE
CZ
LX
K
RZ
Q
Top 50 most variable protein coding genes
(excludes MT proteins)
13.5
Log2(RPKM)
11
8.75
